# Strength of PLA Components Fabricated with Fused Deposition Technology Using a Desktop 3D Printer as a Function of Geometrical Parameters of the Process

**DOI:** 10.3390/polym10030313

**Published:** 2018-03-13

**Authors:** Vladimir E. Kuznetsov, Alexey N. Solonin, Oleg D. Urzhumtsev, Richard Schilling, Azamat G. Tavitov

**Affiliations:** 1 Department of Physical Metallurgy of Non-Ferrous Metals, National University of Science and Technology “MISIS”, Leninskiy Prospekt 4, NUST MISIS, Moscow 119049, Russia; Solonin@misis.ru (A.N.S.); darikcr@gmail.com (O.D.U.); aztapps@gmail.com (A.G.T.); 2 School of Textiles and Design, Reutlingen University, Alteburgstraße 150, D-72762 Reutlingen, Germany; Richard.Schilling@Reutlingen-University.DE

**Keywords:** additive manufacturing, desktop 3D printing, fused deposition modeling, fused filament fabrication, polylactic acid, anisotropy, interlayer bonds, mechanical strength, digital fabrication

## Abstract

The current paper studies the influence of geometrical parameters of the fused deposition modeling (FDM)—fused filament fabrication (FFF) 3D printing process on printed part strength for open source desktop 3D printers and the most popular material used for that purpose—i.e., polylactic acid (PLA). The study was conducted using a set of different nozzles (0.4, 0.6, and 0.8 mm) and a range of layer heights from the minimum to maximum physical limits of the machine. To assess print strength, a novel assessment method is proposed. A tubular sample is loaded in the weakest direction (across layers) in a three-point bending fixture. Mesostructure evaluation through scanning electronic microscopy (SEM) scans of the samples was used to explain the obtained results. We detected a significant influence of geometric process parameters on sample mesostructure, and consequently, on sample strength.

## 1. Introduction

The method of additive manufacturing of voluminous parts by layered distribution of melted polymer was invented by Scott Crump in 1989 [1]. That was perhaps the first important step towards the democratization of digital additive fabrication technologies. FDM turned out to be a substantially more accessible method of 3D printing than the pioneering stereolithography (SLA) and selective laser sintering (SLS), in terms of both the cost of equipment and the cost of materials processed. By the beginning of the 2000s, Stratasys—the company founded by Crump—must have owned all the intellectual and material resources necessary to build a personal desktop printer [2], yet for a number of reasons products of that kind were not launched by Stratasys. More recently, they have been launched by enthusiasts and open projects—the most important of which was the RepRap initiated by Adrian Bowyer and his colleagues [3,4]. The project declared its aim to develop a 3D printer which could be printed by another 3D printer. Although none of the RepRap printers can fully replicate themselves, the project’s success led to the formation of an ecosystem of desktop digital additive fabrication. The FDM technology under the name of FFF appeared in the mass market.

Open source software and hardware of desktop 3D printers gives full control over the speed and temperature management during the printing process. A reasonable choice of geometrical parameters and the temperature–speed settings on desktop 3D printers in many cases allows better product properties (including higher strength) to be obtained compared to products made using far more expensive proprietary machines documented by research results [5].

The overwhelming majority of products made by desktop printers today are various toys and customized casings for cell phones, but there are also examples of products with serious applications. The most resonating of these are Cody Wilson’s Liberator [6] and the E-nable project [7].

The last decade saw explosive growth in the number of 3D printers, basically caused by the mass-market segment of desktop machines, which led to the appearance of a substantial market of supplies—polymers for printing in the “filament” format with thread diameters of 1.75 and 2.85 mm. Introduced by Stratasys, FDM printing started exclusively with polyacrylonitrile butadiene styrene (ABS); the FFF printing initiated by RepRap started with polycaprolactone (PCL) [4]; but today a variety of materials, including polylactic acid (PLA), polycarbonate (PC), polyetheretherketone (PEEK), and many others can be used as filament material for 3D printing. Meanwhile, despite this variety, the first choice in the majority of applications is currently polylactide (PLA). PLA is favored for its biodegradability, absence of unpleasant odors when heated, and for its overall environmental compatibility in all aspects of its life cycle. Additionally, PLA emits ten times less potentially dangerous ultra-fine particles [8] than ABS.

Nonetheless, the main advantage of polylactide in comparison to alternative polymers used for 3D printing is perhaps the low level of shrinkage and relatively low melting temperature. The former leads to a minimum level of residual stresses in the printed parts, resulting in the absence of deformation and delamination; the latter leads to higher productivity of the printing process. PLA is mostly criticized due to its relatively low durability (less than ABS), but this verdict is not fully justified. Parts made of 3D-printed PLA perform significantly worse than those made of ABS at elevated temperatures; however, the opposite is true at normal ambient temperatures. Due to residual stresses caused by hindered shrinkage of the polymer during the cooling process, a part made of ABS can fail by delamination at minimal or no external loads. Parts made of ABS frequently start to delaminate even before the printing is finished. Thus, 3D-printed parts made of PLA (which is less prone to shrinking) contain less internal stresses and thereby show better mechanical characteristics, shown for example in [5], where the mean tensile strength of the samples is measured to be 28.5 MPa for ABS and 56.6 MPa for PLA.

Parts obtained by FDM (FFF) have an explicit anisotropy of mechanical properties, caused by the process characteristics of depositing layers of fine threads of molten thermoplastic material. The strength of FDM (FFF) parts across the layers (in the Z-direction) is significantly lower than the strength along the threads (X- and Y-direction, respectively), sometimes by an order of magnitude. In the majority of studies investigating the influence of various parameters on the strength of FDM (FFF) technologies [5,9,10,11], tensile properties are measured on flat samples oriented horizontally during the printing process to be researched (i.e., with the smallest dimension in the Z axis). Thus, during the tests samples are stressed alongside the printed strands, of which the shell of the sample consists (i.e., in the direction where the sample exhibits its maximum strength).

Interfaces between layers of FDM (FFF) prints are morphologically very similar to so-called weld or knit lines in injection-molded parts. Earlier studies on these interfaces show that their mechanical performance is substantially inferior to that of homogeneous parts made of the same material and to the bulk properties, respectively [12].

Attempts to significantly improve the strength of 3D printed parts therefore need to focus on the Z axis. In this respect, the most important parameter is not the strength of the filament material itself, but the strength and stability of the bonds between the layers of the sample, which in turn are largely determined by the printing parameters. Research on the influence of process parameters on the strength of samples in the Z-axis is hitherto scarce. 

The objective of this paper is to study the bonding strength between adjacent layers as a function of geometrical parameters of the FDM (FFF) process.

## 2. Experimental Methods

There is currently no standard procedure for testing specimens fabricated on FDM (FFF) printers. In the majority of papers observed [5,9,10,11], the testing standards used have been developed for polymer samples produced by molding or by machining or die cutting out of monolithic sheet or slab [13], and consequently do not consider the specifics of samples made of fused filament threads. 

Q. Sun et al. [14] propose a method of measuring the flexural strength of laminated sheets bonded with glue [15], which can be viewed as a more appropriate approach, whilst still being limited in applicability.

In the absence of generally accepted standard procedures, an alternative method is proposed: testing the whole part in the least strong direction. The strength of the boundary between two layers being the factor limiting the strength of FDM (FFF) specimen, bending tests seem more adequate than tensile ones. This is also true considering typical loads on printed parts. Among the various procedures of measuring flexural strength, the three-point-bending test is viewed as the most appropriate. The sample geometry was selected in order to allow varying the fabrication process parameters in a wide range. The model is decomposed by printer software into upper and lower planes, shell mimicking the shape of the model, and infill inside the shell. All the practical experience in 3D printing shows that for an improvement of mechanical strength, it is first necessary to invest printing time and material into the shell rather than into the infill. When the shell is printed, the filament strand is laid down parallel to the preceding one, ensuring maximum contact area between the layers. For this reason, the samples for the method presented consist exclusively of a shell. All the samples prepared for this paper had the same dimensions and shape. 

### 2.1. Samples Fabrication

**Figure 1:**
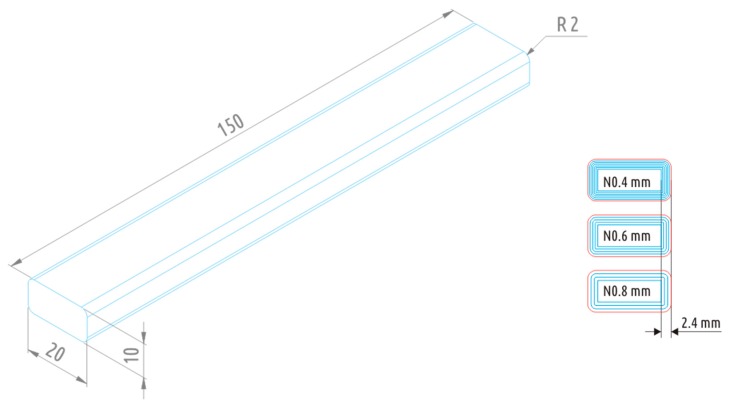
Solid model for fabrication of the testing sample and cross-sections of samples printed with 0.4, 0.6, and 0.8 mm nozzles.

The sample shape chosen was a tube of a rectangular cross-section of 10 × 20 mm^2^ with rounded corners of 2 mm outer radius, with wall thickness of 2.4 and 150 mm in length. The file used initially for printing was a solid model of a parallelepiped of 10 × 20 × 150 mm^3^ with rounded (*R* = 2 mm) long ribs (Figure 1).

The samples were printed in an upright position (i.e., with the 150 mm dimension along the Z-axis); the tubular geometry was achieved by setting the “fill density” parameter in the printer software to 0 and by avoiding the upper and lower covers (solid infill top and solid infill bottom). The wall thickness was preset by the parameter “shell thickness”.

Three different nozzles were used for producing the samples of 0.4, 0.6, and 0.8 mm in diameter, respectively. The shell was printed with six, four, and three rounds of the respective nozzle; for FDM (FFF), shell thickness usually is an integer multiple of nozzle diameter, and accordingly, the shell is formed by one or several perimeters.

The layer thickness of the prints varied between 0.06 and 0.6 mm; for each combination of nozzle diameter and layer thickness, at least three samples were produced. Irrespective of the nozzle used, the layer thickness chosen, and the type of printer, all the samples were printed with a constant linear speed of 25 mm/s, which obviously caused a wide variation in volumetric speed, which in turn is calculated as the volume of plastic extruded through the nozzle per unit of time. The 0.4-mm nozzle was used on a serial Ultimaker 2 printer (Ultimaker B.V., Geldermalsen, The Netherlands), whilst the 0.6 and 0.8 mm nozzles were used in an Ultimaker 2 with a modified heating unit known as the “Olsson Block” (Anders Olsson, The Netherlands) [16], allowing use of replaceable nozzles with varying diameters, and a heating element with increased heating power (ca. 35 W). Both printers were fed with a filament 2.85 mm in diameter.

In all experiments, during the first stage, PLA filament from a single supplier (REC, Moscow, Russia) of one color (turquoise) and from the same batch was used. It was also necessary to generalize intermediate results by showing that parameter optimization results obtained for one PLA-based filament can be applied to prints using different PLA-based filaments. To answer this question, a set of additional experiments was performed to estimate the behavior of parts printed from other types of PLA. In addition to the turquoise PLA by REC, there were three other filaments investigated: colorless (natural) PLA from the same manufacturer (REC), as well as colorless (natural) and black filament by Verbatim (produced in China, Verbatim GmbH, Eschborn, Germany). The samples were printed under the same conditions used in the first part of the study. There were two nozzles used (0.4 and 0.6 mm); the first was utilized to print samples with 0.06 and 0.2 mm layer height, the second for samples with 0.1 and 0.4 mm layer height. 

### 2.2. Mechanical Strength Examination

The printed samples were tested with a universal electromechanical testing machine (OOO Tochpribor, Ivanovo, Russia) with a test rig for three-point bending. The samples rested on two cylindrical supports of 30 mm diameter with a distance of 100 mm between the centers. The load was put on the middle of the sample between the supports by a cylinder 40 mm in diameter.

The tests were carried out with the crosshead of the tensile tester moving at a speed of 10 mm/min. During the test, the deformation and load on the sample were recorded, with the initial load for stabilizing the sample set at 5 N. The key parameter was the maximum load measured before the samples failed (UFL). The broken samples were visually checked to identify the type of failure they experienced during the test. There are two primary types of failure—in the bulk (across the layers) or on the interface between layers. Cross-section mesostructures of some of the broken samples were investigated using SEM on a Tescan Vega 3 LMH microscope microscope (TESCAN Brno, s.r.o., Brno-Kohoutovice, Czech Republic).

For a more general discussion of the results, the normal stresses in the samples at the moment of failure (UFS) were calculated. The stresses were calculated by dividing the maximum moment (*M*) by the section modulus (*W*):

$$\sigma_{max}=\frac{M}{W},\;\mathrm{MPa}.$$

The maximum moment for testing samples at distance between the supports of 100 mm:

$$M=\frac{F\times l}{4}=0.025F,\;N\cdot m,$$

where *F* is the load in N at the moment of failure.

The section moment is calculated by using formula:

$$W=\frac{2I}{h},\;m^{3},$$

where *I* is the second moment of the cross-section, calculated with the built-in app of SolidWorks to be 1416.89 mm^4^.

Thus, the maximum normal stress in the sample can be calculated from the load by

$$\sigma_{max}=0.088F,$$

where *F* is given in N and $\sigma_{max}$ is in MPa.

## 3. Results and Discussion

### 3.1. Influence of Geometrical Parameters

**Figure 2:**
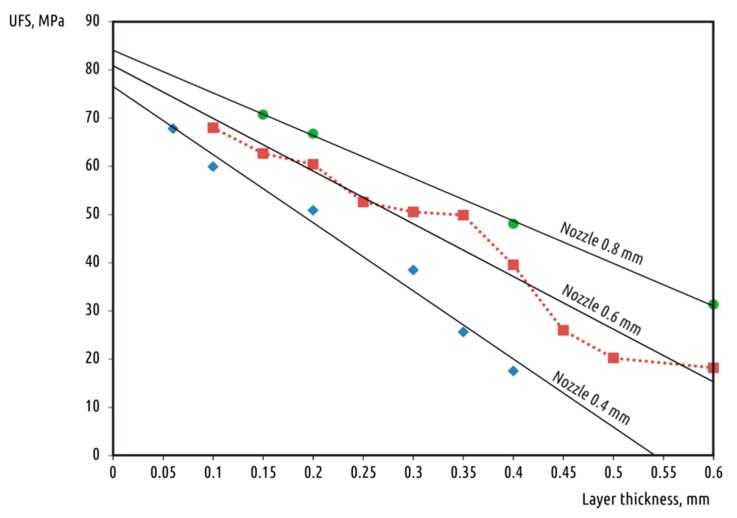
Ultimate flexural strength (UFS) of tested samples versus deposited layer height for all nozzles used.

Experimental data (Table 1) showed a clear dependence of the parameter being varied on sample strength (Figure 2). Results suggest that increasing the nozzle diameter not only reduces printing time, but also increases sample strength. For each of the nozzles used, strength degraded as layer thickness increased. That dependency was observed over the whole range of parameters investigated. Each nozzle diameter allowed for varying layer heights across a wide range, up to theoretically possible limits for a given machine. The minimal layer height implemented for the 0.4-mm nozzle was 0.06 mm, which is the absolute minimum proposed by the printer manufacturer for the Ultimaker 2 machine. The maximum layer thickness values tested were equal to nozzle diameters for 0.4- and 0.6-mm nozzles. However, using layer thickness more than 80% of nozzle diameter seems impractical; thicker layers were considered only to complete the image.

The dependency of strength w.r.t. layer height can be approximated by the linear function proposed in Figure 2. 

The current paper might be the first publication presenting strength data for PLA samples printed with 0.8- and 0.6-mm nozzles. However, data for samples printed with 0.4-mm nozzles conflict with results of previous research [10]. That study claims that increasing layer height also increased sample strength instead of a drastic reduction: “In upright samples, tensile and flexural strength increased as layer thickness increased”. The authors of that research also indicate the correspondence of their results with previous studies [11]. However, experiment settings in [10,11] have significant differences that impede direct comparison. For example, [11] scrutinizes ABS samples printed on a proprietary Stratasys Vantage machine. The printing strategy (“perimeter and interior”, equal to “shell and infill” in modern open source printer and slicer terminology) was different from the study in [10], where the shell constituted the whole sample volume. The results of [11] are ambiguous, as tensile strength is increased along with layer thickness, while the flexural strength tests do not exhibit that relation clearly: increasing layer thickness can both increase and reduce flexural strength, depending on other settings.

A comparison of absolute strength values for three-point bending was performed to discover differences between the current approach and previous works. The maximal UFS value in [10] is 34.5 MPa for layer thickness range between 0.06 and 0.24 mm for 20 mm/s printing speed (the minimal value is 25.1 with the mean of 30.42). The current research obtained UFS values in the range of 38.7 to 67.8 MPa for 0.06–0.3 mm layer thickness (0.4-mm nozzle). For reference, bulk PLA flexural strength lies in the range of 83 to 108 MPa [17]. Such a big difference between results of the current study and the results obtained in [10] can only be explained by different experimental setups. The setting created by the Spanish research group suggests the following hypothesis. ASTM standard proposes a sample with very large length-to-cross-section (width multiplied by thickness) ratio. Printing in an upright position means very large height to bottom surface area ratio, resulting in a tall and unstable object. Nozzle movement—even given the reduced 20 mm/s speed—causes oscillations of the sample, thus reducing surface quality and interlayer cohesion. If that suggestion is correct, the results obtained in [10] are not applicable in a common printing setup for stable samples.

Strength degradation with increasing layer height was also registered in [18], although the object of study was different, as ABS-printed samples were used with only two options for layer height. 

**Figure 3:**
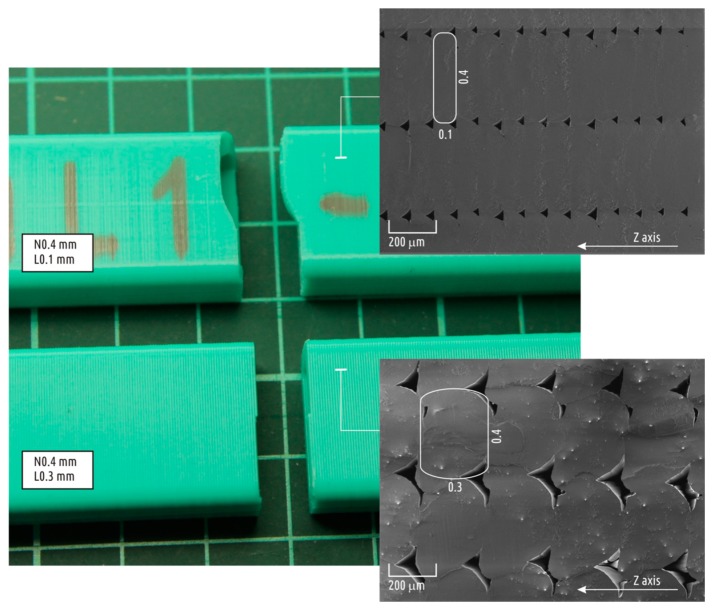
Appearance (**left**) of typical samples with relatively small (**top**) and relatively large (**bottom**) layer thickness, and SEM images of their cross-sections (**right**).

The sample printed with relatively thick layers shows that breakage occurred at the junction between layers (Figure 3 bottom), whereas the crack in the 0.1 mm sample (Figure 3 top) was not planar. The X–Z axes cross-sections (right side of Figure 3) of these samples were analyzed using a scanning electronic microscope (SEM), and the images obtained clearly explain the dependencies discovered.

Even when printing the shell, FDM (FFF) material was still far from being monolithic. The cross-section clearly displays individual threads that constitute the part, as well as voids between them. These voids significantly reduce the effective sample cross-sections in X–Y axes, making it lower than the calculated value. Thus, the calculated UFS values have no physical meaning, while being a virtual measure solely for comparison purposes. SEM scans show that gap areas increase along with layer height, and the difference between actual cross-section area and sample cross-section used for calculations will rise as well. That is the proposed explanation for why sample strength decays with layer height increase.

Increase in interlayer cohesion with nozzle diameter increase can be explained by the following. When single thread thickness is increased, a shell of the same thickness will be made of a smaller number of individual threads, while voids between them remain of comparable size with constant layer height, and thus the print will contain less total space between these threads.

**Figure 4:**
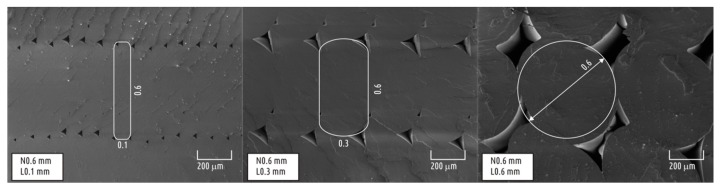
Transformation of an individual thread cross-section with layer thickness increase: idealized layer thread shape overlaid on SEM images.

The data obtained can be generalized with the following formulation: the higher the ratio of nozzle diameter to layer height, the more the individual thread of the deposed filament resembles an ideal circle. The smaller the layer height, the more the cross-section resembles an elongated rectangle. That could be illustrated by images of the cross-sections of samples printed with 0.6-mm nozzles and with minimum (0.1 mm), mean (0.3 mm), and maximum (0.6 mm) layer thickness (Figure 4). 

**Figure 5:**
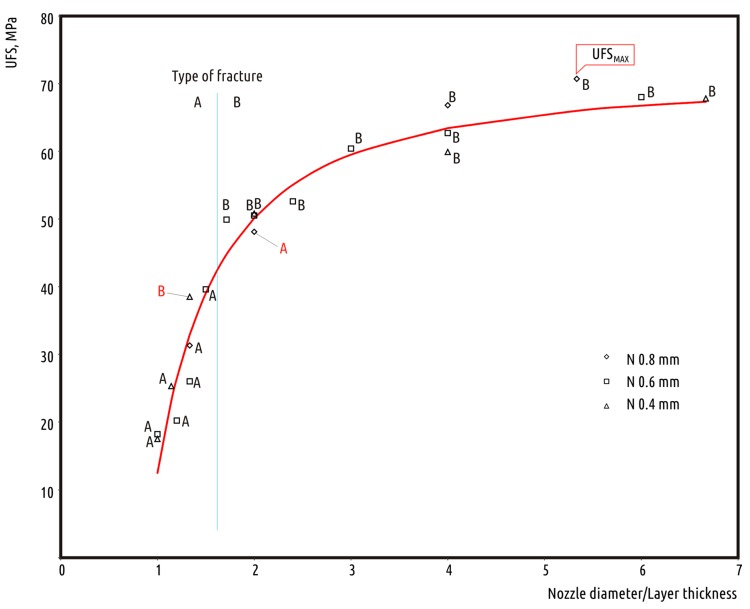
Ultimate flexural strength of the tested samples versus nozzle diameter-to-layer height ratio.

Thus, the most important factor that defined the strength of the resulting part was the interlayer contact surface area, which is defined by nozzle diameter to layer height ratio. The following scatter diagram in the UFS/(N/L) coordinate system shows that all the observations can be fitted with a single curve (Figure 5).

The curve fitting the experimental data with adequate accuracy may be represented by formula:

*UFS* = *UFS_MAX_* − 58.19/(*r*^1.5^),

where *UFS_MAX_* is the maximum UFS observed in the experiment, *r* is the nozzle diameter-to-layer height ratio, and *UFS* is the resulting part strength. 

This formula was obtained using curve fitting approach with Scikit-learn package implementation in Python as a result of optimization for the following formula:

*UFS* = a − b/(*r*^c^).

Unconstrained optimization resulted in *R*^2^ score equal to 0.958, and coefficient *a* was calculated slightly below *UFS_MAX_*. As the value resembled bulk material strength or maximum *UFS* value obtained, it was decided that for clarity purposes the resulting formula will use *UFS_MAX_* as *a*. *R*^2^ score reduced insignificantly to 0.954, which seemed a perfect result.

From a practical point of view, it might be more useful to just divide the scatterplot into three zones labeled as the “unadvised”, “optimal”, and “rigorous” ranges in terms of resulting part strength. Printing with a nozzle-to-layer height ratio (*N/L*) between 1 and 2 can only be advised for making low-duty prints. These can be parts not bearing physical loads, and for the cases when printing time is more important than load capacity. The range of *N/L* ratio between 2 and 4 can be considered optimal for printing functional parts. Varying geometrical parameters allows for finding a compromise between printing time and part strength. Raising the ratio above 4 can only be recommended for the fabrication of crucial parts, when printing time becomes insignificant.

The (*N*/*L*) ratio not only determines the part strength quantitatively, but also qualitatively. The ratio value of about 1.6 divides all the observations—except for two outliers marked with red color in Figure 5—into two types of failure: across the layers (A) and in the bulk (B).

### 3.2. Influence of Filament Material on Print Strength

The current subsection should be prefaced with the following Disclaimer:

Filament quality is a multidimensional notion which defines different parameters, from uniform spooling to biodegradability. The sample strength obtained when using a specific filament with a specific printing configuration and parameters is just one of many quality measures, and increasing the sample strength might adversely affect other parameters. The current study concentrated on analyzing opportunities to increase sample strength across layers. We cannot claim that some of the filaments are better or worse than others; we just showed that samples printed using different filament from various suppliers at parameters indicated exhibited different strength.

**Figure 6:**
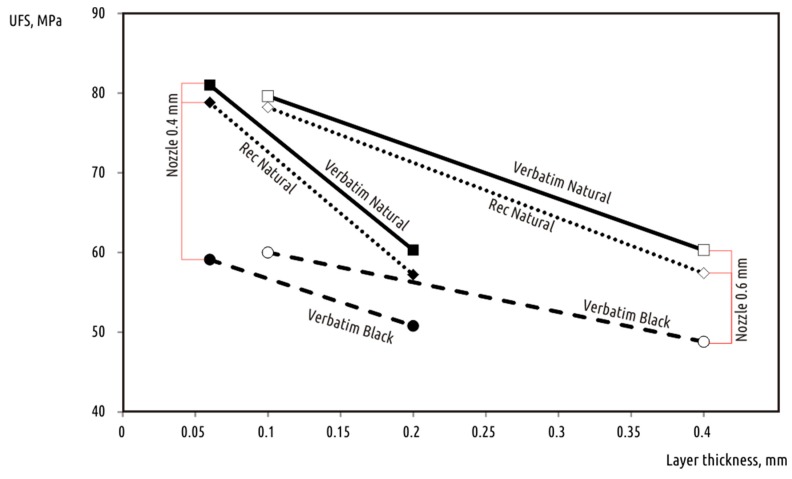
Ultimate flexural strength versus deposited layer height for additional materials tested.

As flexural strength results (Table 2) show, different plastics may significantly vary in strength, but the general dependency (positive correlation between sample strength and nozzle diameter, and negative for layer thickness) remains the same (Figure 6). The highest UFS values were obtained for transparent filaments. This is in line with [19], where natural filament showed slightly better strength than black, blue, gray, or white. That suggests that any pigments added to PLA base reduce the printed part strength to some extent. 

## 4. Conclusions

This paper offers a new methodology for researching the influence of material and process parameters on the mechanical properties of parts printed with FDM (FFF) technology. That methodology allows more adequate results to be obtained than with the most common research approaches based on standards which are not applicable to FDM (FFF) printing technology.

The study showed that limiting printed part strength may be significantly increased by adjusting basic geometry parameters of the process. This should be true for most 3D printing filaments based on PLA for desktop printers available, including all filaments tested.

The layer height had the greatest influence on intra-layer cohesion. Part strength decreased along with layer height increase for all nozzles investigated (0.4, 0.6, and 0.8 mm) over the whole range of layer height values tested. For the samples under study, the decrease of strength when changing layer height from minimum to maximum was about 3.5 times.

Nozzle diameter also had a significant influence on interlayer cohesion. Given constant layer height, printing with a larger nozzle resulted in increased strength. The advantages of larger nozzles became even more evident with greater layer thickness.

The mechanical properties of a part obtained by using the FDM (FFF) process depended on the filament used, and may vary between different manufacturers, colors, lots, and even spools within a single lot. However, dependencies obtained should be generalizable for all PLA-based filaments.

The best combinations of printing conditions allowed for obtaining interlayer cohesion (the strength of a part loaded orthogonally to layer boundaries) close to the known parameters of bulk PLA material. This partially resolves the anisotropy problem for FDM (FFF) printed parts.

## Figures and Tables

**Table 1:** Experimental data on the influence of geometrical parameters of 3D printing (nozzle diameter and layer thickness) on the fabricated components’ strength.

Code	Nozzle Diameter, mm	Layer Thickness, mm	Volumetric Printing Speed, mm^3^/s	Fracture Force ^1^, N	Ultimate Fracture Strength, MPa	Type of Fracture ^2^
N8L15	0.8	0.15	3	802 (27)	70.7	B
N8L2	0.8	0.2	4	757 (35)	66.8	B
N8L4	0.8	0.4	8	545 (72)	48.1	A
N8L6	0.8	0.6	12	355 (27)	31.3	A
N6L1	0.6	0.1	1.5	771 (17)	68.0	B
N6L15	0.6	0.15	2.2	710 (49)	62.7	B
N6L2	0.6	0.2	3	685 (9)	60.4	B
N6L25	0.6	0.25	3.8	596 (30)	52.6	B
N6L3	0.6	0.3	4.5	573 (51)	50.5	B
N6L35	0.6	0.35	5.2	565 (34)	49.9	B
N6L4	0.6	0.4	6	448 (13)	39.6	A
N6L45	0.6	0.45	6.8	294 (4)	26.0	A
N6L5	0.6	0.5	7.5	229 (21)	20.2	A
N6L6	0.6	0.6	9	207 (12)	18.2	A
N4L06	0.4	0.06	0.6	768 (7)	67.8	B
N4L1	0.4	0.1	1	679 (33)	59.9	B
N4L2	0.4	0.2	2	576 (44)	50.8	B
N4L3	0.4	0.3	3	436 (59)	38.5	B
N4L35	0.4	0.35	3.5	290 (18)	25.6	A
N4L4	0.4	0.4	4	198 (24)	17.5	A

^1^ Value in brackets is a standard deviation; ^2^ A—on the interface between layers, B—in the bulk (across the layers).

**Table 2:** Experimental data on the influence of geometrical parameters (nozzle diameter and layer thickness) on strength of components fabricated from different polylactic acid (PLA) filaments.

Vendor	Color	Nozzle Diameter, mm	Layer Thickness, mm	Fracture Force ^1^, N	Ultimate Fracture Strength, MPa
REC	Natural	0.6	0.1	886 (19)	78.2
REC	Natural	0.6	0.4	651 (6)	57.4
Verbatim	Natural	0.6	0.1	902 (38)	79.6
Verbatim	Natural	0.6	0.4	683 (29)	60.3
Verbatim	Black	0.6	0.1	680 (38)	60.0
Verbatim	Black	0.6	0.4	553 (44)	48.8
REC	Natural	0.4	0.06	893 (20)	78.8
REC	Natural	0.4	0.2	648 (40)	57.2
Verbatim	Natural	0.4	0.06	918 (37)	81.0
Verbatim	Natural	0.4	0.2	684 (16)	60.3
Verbatim	Black	0.4	0.06	670 (38)	59.1
Verbatim	Black	0.4	0.2	576 (61)	50.8

^1^ Value in brackets is a standard deviation.
